# Benefits of BV-AVD (Brentuximab vedotin, doxorubicin, vinblastine, and dacarbazine) versus ABVD (doxorubicin hydrochloride, bleomycin sulfate, vinblastine sulfate, dacarbazine) in patients with advanced-stage Hodgkin's lymphoma: an analysis of the ECHELON 1 trial by the GATLA group using the Delphi Method

**DOI:** 10.1016/j.htct.2024.07.007

**Published:** 2024-09-25

**Authors:** Astrid Pavlovsky, Juan Ignacio Garcia Altuve, Amalia Cerutti, Lorena Fiad, Nicolás Kurgansky, Fernando Warley, Florencia Negri Aranguren

**Affiliations:** a Centro de Hematologia Pavlovsky, Buenos Aires, Argentina; b FUNDALEU, Buenos Aires, Argentina; c Centro de Educación Médica e Investigaciones Clínicas (CEMIC), Buenos Aires, Argentina; d Sanatorio Británico de Rosario, Santa Fé, Argentina; e Hospital Italiano La Plata, Buenos Aires, Argentina; f DOCTUS, Buenos Aires, Argentina; g Hospital Italiano de Bueno Aires, Buenos Aires, Argentina; h Instituto Privado de Hematologia y Hemoterapia, Entre Ríos, Argentina

**Keywords:** Hodgkin's lymphoma, Delphi method, Advanced-stage, Brentuximab

## Abstract

**Introduction:**

The BV-AVD (Brentuximab vedotin, doxorubicin, vinblastine, and dacarbazine) combination for first-line treatment of advanced stage Hodgkin's lymphoma has been approved by regulatory authorities and included in international guidelines. However, several factors influence its incorporation as standard of care.

**Materials and Methods:**

A group of experts from different institutions was identified and, using the Delphi method, an analysis of the results of the ECHELON 1 trial for the indication of BV-AVD over ABVD (doxorubicin hydrochloride, bleomycin sulfate, vinblastine sulfate, dacarbazine) in patients with Hodgkin's lymphoma Stages III and IV in Argentina was done. The clinical and academic experience of the authors and the context of the Argentine healthcare system were considered.

**Results and discussion:**

Seven statements on general aspects of the management of Hodgkin's lymphoma and nine on specific aspects related to the use of BV-AVD over ABVD reached a consensus of agreement. There was a strong expert consensus in favor of indicating BV-AVD in the presence of extranodal disease or pulmonary disease. Moderate to severe neuropathy, pregnancy and drug allergy were considered absolute contraindications to prescribe BV.

**Conclusions:**

The authors agreed that BV-AVD could be considered a new treatment option in high-risk patients. However health system-dependent factors (such as high cost, lack of availability, reimbursement difficulties, irregular delivery, and issues with granulocyte-colony stimulating factor availability) could pose limitations for this prescription. While awaiting new data from clinical trials and real-world studies, these recommendations can represent a useful tool for hematologists in different parts of the world.

## Introduction

Classical Hodgkin's lymphoma (HL) is a hematological malignancy predominantly affecting young adults aged 15 to 30 years, with a secondary peak observed after the age of 55 years.^1^ Over the past decades, the integration of combined radiotherapy (RT) and chemotherapy, notably the ABVD regimen (doxorubicin, bleomycin, vinblastine, dacarbazine), has substantially enhanced survival rates, soaring from 69 % in 1975 to 98 % in 2020.^2^ Despite these advancements, concerns persist regarding acute and long-term treatment-related toxicities, including bleomycin-induced lung damage and increased mortality compared to the general population.^3^

Recent studies, such as the RATHL, AHL2011, and HD18 trials, have shifted the paradigm towards positron emission tomography/computed tomography (PET/CT)-adapted treatment strategies, demonstrating safety in de-escalating treatment for patients with negative interim PET/CT scans and intensifying therapy for those with positive findings.^4^, ^5^, ^6^ Notably, the GATLA LH-05 trial assessed PET/CT-guided therapy in HL patients, revealing promising outcomes with three-year event-free survival (EFS) of 90 % in early-stage disease and 72 % in advanced stages.^7^^,^^8^ These findings prompted the integration of PET3-adapted therapy into routine practice for advanced-stage HL.

The ECHELON-1 trial introduced brentuximab vedotin (BV) in combination with doxorubicin, vinblastine, and dacarbazine (BV + AVD) as a novel frontline regimen for advanced-stage HL, achieving significant improvements in progression-free survival (PFS) and overall survival (OS) compared to ABVD.^9^^,^^10^ While BV + AVD is endorsed by regulatory authorities and guidelines, its adoption as a standard treatment warrants careful consideration due to the absence of interim PET/CT guidance, associated toxicities, and its economic implications.^11^

In light of these developments, a collaborative effort among seven investigators from Argentina aims to provide comprehensive recommendations on the use of BV + AVD in advanced-stage HL patients previously managed with PET3-guided ABVD therapy. Drawing from their expertise, published data, and regional considerations, these recommendations seek to optimize treatment outcomes across diverse clinical settings.

## Material and methods

Since the 1990s, the introduction of evidence-based medicine approaches has enabled the optimization of clinical practice guidelines and recommendations. However, the accurate interpretation of available evidence, and its adaptation into a sociocultural environment different from the original one, hinders implementation in everyday practice. For this reason, the Delphi expert consensus method is considered a valid and reliable strategy.^12^This technique consists of a structured process involving several stages of questions (‘rounds’) which are held until a group of experts reaches consensus through statistics and controlled feedback.^13^^,^^14^ Data on previous experiences with its use in Argentina are available.^15^^,^^16^

A panel of experts from Argentina, members from the GATLA (*Grupo Argentino de Tratamiento de la Leucemia Aguda*) cooperative group with long-standing experience in the management of advanced-stage HL, were invited to analyze the results of the ECHELON 1 trial.

Two questionnaires were prepared based on evidence from the most relevant biomedical databases. The first questionnaire was related to general treatment aspects and consisted of 24 questions (13 with response options using a 10-point Likert scale, five with a polyatomic response and six open-text questions). The second questionnaire addressed the use of BV as first-line treatment for patients with Stage III-IV HL and consisted of 18 questions (11 with response options using a 10-point Likert scale, one with a polyatomic response and six open-text questions).

The questionnaires were shared with the panel of experts via a digital platform in order to control blinding and anonymity of responses. A strong (very high) expert consensus was defined as ≥80 % agreement and expert consensus was reached when the degree of agreement was ≥70 %, with an abstention rate below 20 %. For questions where no consensus was reached in the first round, some answers were rephrased based on the suggestions from the expert panel. These questions were submitted again for examination during a second round. A measure of stability to conclude the consultation was determined when 70 % of experts did not change their answer in consecutive rounds.

Finally, a draft including the resulting recommendations for review and approval by all the experts was created after a closing virtual meeting.

Although economic aspects were assessed based on the available evidence, the clinical and academic experience of the authors, and the variable context of the healthcare system in Argentina at the time of the creation of these recommendations, a formal pharmacoeconomic analysis was excluded from this document and will be reserved for future research by field specialists.

This study was supported by an unrestricted grant provided by Takeda. The sponsor had no active role in the selection of the authors, opinions and final statements of this paper.

## Results and discussion

### Part 1 – general aspects

[**A1**] Based on the available evidence and the experience of this group of experts, the panel agrees that the following factors should be essentially considered during decision-making in the everyday practice for the initial assessment of patients with Stage III/IV HL before any first-line treatment:

**Table:** 

PET/CT or CT of neck, chest, abdomen, and pelvis in patients without a baseline PET/CT available (*)
IPS score (**)
Geriatric assessment in older adults (*)
Echocardiogram (*)
Assessment of pulmonary reserve (*)
Fertility and contraception counseling (**)

(*) Strong expert consensus; (**) expert consensus

[**A2**] Based on the available evidence and the experience of this group of experts, the panel believes that these factors are critical when selecting first-line treatment in patients with advanced LH:

**Table:** 

Patient age (*)
IPS score (*)
Presence of neurological and/or pulmonary comorbidities (*)
Availability of PET/CT (*)
Patient access to treatment facilities (distance, time) (**)
Access to drugs through the patient's health insurance and the institution (*)

(*)Strong expert consensus; (**) expert consensus

[**A3**] Based on the available evidence and the experience of this group of experts, the panel considers that interim PET/CT-adapted ABVD or BV-AVD are first-line treatment options for patients with Stage III/IV HL, depending on the availability of drugs and diagnostic methods. This group of experts does not routinely use BEACOPP (bleomycin, etoposide, doxorubicin, cyclophosphamide, vincristine, procarbazine, and prednisone) as first-line treatment for advanced stages of HL; for this reason, BEACOPP was not considered in this analysis.

### Strong expert consensus recommendation/statement

Previous trials demonstrated the effectiveness of ABVD for the treatment of Stage III/IV HL.^4^^,^^5^^,^^17^^,^^18^ The RATHL study proved that removing bleomycin (and continuing treatment with AVD) is safe in patients with negative PET/CT after two cycles of therapy with ABVD. The three-year PFS and OS rates in the AVD arm were 84.5 %, (95 % confidence interval [95 % CI]: 80.7–87.5) and 97.6 % (95 % CI: 95.6–98.7), respectively, and 85.7 % (95 % CI: 88.1–88.6) and 97.2 % (95 % CI: 95.1–98.4) in the ABVD arm. As a result, this approach has now been included in treatment guidelines. This same study tested the strategy of escalating to BEACOPP-14 or BEACOPP_ESC_ after a positive interim PET/CT (at the investigator's discretion), with an adequate toxicity profile and a three-year PFS of 67.5 % (95 % CI: 59.7–74.2). ^4^

Since the advent of ABVD, ECHELON-1 was the first trial to demonstrate improvements in OS with BV-AVD with an OS of 93.9 % (95 % CI: 91.6–95.5) for the BV-AVD arm versus 89.4 % (95 % CI: 86.6–91.7) for the ABVD arm. ^9^

[**A4**] Based on the available evidence and the experience of this group of experts, the panel recommends PET/CT-based staging for all patients with Stage III/IV HL.

### Strong expert consensus recommendation/statement

PET/CT has proven to be more accurate and sensitive than conventional CT for initial staging in several trials. As shown by Hutchings et al., the greater sensitivity of PET/CT is particularly improved for spleen and bone marrow involvement. ^15^ These data are also consistent with other papers reporting a higher sensitivity for PET/CT in contrast to conventional CT, thereby leading to a change from 10 % to 40 % in staging, as well as changes in treatment intensity in 20 % of these patients. 19, 20, 21

Finally, when planning RT, PET/CT-guided staging can also significantly influence the extent of the involved field, compared with RT planning based on CT alone. ^4^

[**A5**] Based on the available evidence and the experience of this group of experts, the panel recommends an interim PET/CT (preferably at 12–14 days) for all patients with Stage III/IV HL who initially received 2–3 cycles of ABVD.

### Strong expert consensus recommendation/statement

The RATHL, GATLA LH-05, FIL HD0801 and Gallamini et al. trials support the proven benefit of interim PET/CT-adapted ABVD treatment.^4^^,^^5^^,^^22^^,^^23^ Interim PET/CT scanning should be performed >10 days after the preceding dose of chemotherapy, i.e., as close as possible to the next cycle of cancer treatment.^24^

PET/CT scans should not be performed at 7–10 days after chemotherapy in order to avoid the peak inflammatory response during this period.^23^

[**A6**] Based on the available evidence and the experience of this group of experts, the panel suggests treatment escalation in patients with Stage III/IV HL who initially received two cycles of ABVD and have a Deauville score (DS) ≥4 in their interim PET/CT.

### Expert consensus recommendation/statement

As previously described, the RATHL study^4^ escalated treatment to BEACOPP/BEACOPP_ESC_ in patients with positive interim PET/CT scans (DS 4–5) and achieved a three-year PFS rate of 71 %. A similar BEACOPP escalation strategy for patients with positive interim PET/CT scans was evaluated by the US Intergroup trial S0186 ^17^^,^^25^ and by the Italian Group GITIL/FIL HD 06074,^26^ with PFS rates of 65 % and 60 %, respectively. In the approach used by the Italian Group (HD0801), patients were escalated to salvage therapy and consolidated with an autologous transplant, resulting in a two-year PFS rate of 75 %. ^27^ Although this strategy is used and accepted in high-complexity centers, most institutions still continue to use ABVD for six cycles due to the intensity of BEACOPP_ESC_, or choose to escalate to less intensive regimens and with fewer complications than BEACOPP.

The protocols that mandated shifting patients onto more intensive regimens following an interim PET/CT seem to have shown better outcomes compared with other protocols similar to the one published by Gallamini et al., where patients with a positive interim PET/CT completed six cycles of ABVD with a two-year PFS of 12.8 %, or the GATLA LH-05 trial, in which patients with a positive interim PET/CT continued treatment with ABVD + RT and achieved a three-year PFS rate of 58 %. ^3^^,^^28^

[**A7**] Based on the available evidence and the experience of this group of experts, the panel recommends performing a new biopsy in all patients with Stage III/IV HL who initially received six cycles of treatment and had an end-of-treatment PET/CT DS of 4–5.

### Strong expert consensus recommendation/statement

An end-of-treatment PET/CT DS of 4–5 is indicative of treatment failure; patients are deemed as primary refractory, prescribed with salvage therapy and eventually consolidated with autologous hematopoietic stem cell transplantation. Histologic confirmation with a new biopsy of areas with hypermetabolic activity is recommended before making this decision. This confirmatory biopsy may be avoided in the case of poorly accessible sites remaining PET/CT positive from baseline after proper discussion with a multidisciplinary team and the patient. ^11^

### Part 2 – specific aspects

[**B1**] Based on the available evidence and the experience of this group of experts, factors including comorbid lung disease and extranodal involvement strongly influence the decision to prescribe BV-AVD versus ABVD as first-line therapy.

### Strong expert consensus recommendation/statement

Comorbid lung disease should be carefully considered, as bleomycin-induced lung injury, especially at the interstitial level, was reported in 7 % of patients from the ABVD arm versus 2 % of patients receiving BV-AVD.

The benefit of adding BV to front-line therapy was greater in patients with Stage IV disease and was even higher among patients with extranodal involvement in more than one site. ^6^

[**B2**] Based on the available evidence and the experience of this group of experts, factors including age, the international prognostic score (IPS), and the presence of mild peripheral neuropathy (of any cause) influence the decision to prescribe BV-AVD versus ABVD as first-line therapy.

### Expert consensus recommendation/statement

***Good practice point:*** As defined by the panel, this treatment is contraindicated in patients with moderate-to-severe peripheral neuropathy (B4 recommendation).

Age might be a highly relevant factor when prescribing BV-AVD. On one hand, a significant benefit in OS was observed in under 60-year-old patients (hazard ratio [HR] = 0.51; 95 % CI: 0.29–0.89) compared with over 60-year-old patients (HR: 0.83; 95 % CI: 0.47–1.47). Although this may be due to the small sample size in the subgroup of patients aged over 60, this difference in the benefit and the higher risk of associated complications (especially increased bone marrow toxicity) should be considered when selecting the most adequate therapy.

In a subgroup analysis, no statistically significant benefits were reported in the PFS of over 60-year-old patients with BV-AVD. These patients experienced greater toxicity compared with patients aged under 60. In addition, BV-AVD was associated with a higher incidence of neuropathy and febrile neutropenia, although the incidence of pulmonary toxicity was lower compared with ABVD. ^29^

The subgroup analysis also reported benefits in the IPS. Importantly, IPS was a stratification factor at randomization, thereby ensuring a balanced patient distribution with several risk groups within the score. In terms of outcomes, an IPS of 4–7 had a HR of 0.48 (95 % CI: 0.26–0.88), whereas groups with an IPS of 2/3 and 0/1 had little or no benefit at all (HR: 0.62; 95 % CI: 0.33–1.14 and HR: 0.97; 95 % CI: 0.34–2.77, respectively).

In the ECHELON-1 trial, exclusion criteria included sensory and/or motor peripheral neuropathy. Peripheral neuropathy occurred in 67 % of patients in the BV arm and 67 % resolved without sequelae. For this reason, current evidence on the use of BV-AVD does not include patients with a previous history of neuropathy. ^9^

[**B3**] Based on the available evidence and the experience of this group of experts, gender does not influence the decision to prescribe BV-AVD versus ABVD as first-line therapy.

No statistically significant benefit was reported in PFS (HR: 0.68; 95 % CI: 0.46–1.01) or OS [HR: 0.96; 95 % CI: 0.51–1.80) rates among women receiving BV-AVD versus ABVD, in contrast to male patients (HR: 0.67; 95 % CI: 0.49–0.91 and HR: 0.43; 95 % CI: 0.25–0.73). ^7^ The reason for the difference in mortality between genders remains unknown. Of note, the risk factors assessed in this analysis were not adjusted for confounders.

This group of experts believes that outcomes derived from the subgroup analyses should be interpreted with caution. Further evidence is required in order to make any recommendations on the role of gender as a determining factor when prescribing BV-AVD over ABVD.

[**B4**] Based on the available evidence and the experience of this group of experts, the panel considers that moderate-to-severe peripheral neuropathy, pregnancy, and severe drug allergies are absolute contraindications to first-line therapy with BV-AVD in patients with HL.

### Strong expert consensus recommendation/statement

***Good practice point*:** Neuropathy is considered the most important toxicity in the BV arm, with a significant impact on treatment adherence and greater challenges in patient management.

Patients with sensory or motor peripheral neuropathy, pregnancy, and severe cardiovascular disease were not enrolled in the ECHELON-1 trial, and so its use may not be safe in these patients.

[**B5**] Based on the available evidence and the experience of this group of experts, the panel considers that patients unwilling to accept the risk of neuropathy have a relative contraindication for BV-AVD as first-line therapy.

### Strong expert consensus recommendation/statement

The global incidence of neuropathy may restrict the use of BV-AVD in patients with increased risk of peripheral neuropathy or in patients where this risk is intended to be minimized by preference or working/trade reasons. If treatment is still initiated in this group of patients, close follow-up is recommended for early detection of potential adverse effects.

Although there are no recommendations on their management, the original protocol does include general guidelines on dose modifications and treatment discontinuation for moderate-to-severe events. ^30^

[**B6**] Based on the opinion of this group of experts, BV-AVD may be considered as a new option for the treatment of higher-risk patients.

### Expert consensus recommendation/statement

ECHELON-1 showed improved OS rates in the following subgroups of patients at a higher risk of relapse prior to treatment:^7^

• Stage IV (HR: 0.48; 95 % CI: 0.29–0.80),
• IPS of 4–7 (HR: 0.48; 95 % CI: 0.26–0.88),
• Extranodal involvement in more than one site (HR: 0.30; 95 % CI: 0.14–0.67).

The results of a published subanalysis support the superiority of BV-AVD over ABVD in patients with Stage IV disease and a high IPS. ^31^

We emphasize that subgroup analyses should be interpreted with caution. Given that survival benefits were reported in the entire cohort, we suggest using BV-AVD as a treatment option for all patients with Stage III/IV HL.

[**B7**] Based on the experience of this group of experts, a number of factors related to the healthcare system (such as high costs, unavailability, reimbursement challenges, irregular supply, and problems with granulocyte-colony stimulating factor [G-CSF] availability) may constitute a limitation to the indication of BV-AVD.

### Expert consensus recommendation/statement

Even though ECHELON-1 demonstrated the superiority of BV-AVD versus ABVD in terms of PFS and OS, we believe that some factors associated with the healthcare system might curb its prescription. The classical ABVD schedule is inexpensive, safe, and highly effective, with high OS rates, provided that treatment is initiated early and with adequate periodicity. Cost is a central aspect when prescribing and discussing the use of BV-AVD with payers; this may delay the authorization and interfere with early treatment initiation.

Unavailability may also be a limitation, as BV may not always be readily available after payer approval. This wait may result in delayed initiation of chemotherapy. Reimbursement challenges and irregular supply are closely related to the foregoing limitations; both factors may contribute to treatment delays or interruptions.

Lastly, shortage of G-CSFs, either because they are not authorized, available or supplied, threatens patients’ safety and chemotherapy continuity. In the event of any of these scenarios, treatment with ABVD should be considered.

[**B8**] Based on the available evidence and the experience of this group of experts, under 60-year-old patients with Stage IV disease, extranodal involvement in at least two sites and an IPS of 4–7 may obtain a greater benefit from treatment with BV-AVD.

### Expert consensus recommendation/statement

***Good practice point*:** Based on the available evidence and the experience of this group of experts, the difference in OS between treatments described in the ECHELON-1 trial is both statistically and clinically significant.

The subgroup analysis from ECHELON-1 revealed that the most important advantages of BV-AVD versus ABVD are observed in patients aged under 60, with Stage IV disease, extranodal involvement in at least two sites, and an IPS of 4–7. However, differences between these two treatments were less significant among over 60-year-old female patients with a low IPS. ^32^

[**B9**] Based on the opinion of this group of experts, considering the results of ECHELON-1 at three years and the availability in our practice, the use of interim PET/CT in patients receiving BV-AVD may be categorized as a good practice.

### Strong expert consensus recommendation/statement

***Good practice point*:** Patients who achieve a complete (DS ≤3) or partial (DS 4) metabolic response may continue therapy for up to six cycles; in case of disease progression (DS 5), patients would be switched to another strategy.

### Expert consensus recommendation

In the ECHELON-1 trial, the treatment protocol was not based on the interim PET/CT response. ^6^^,^^7^ In light of the extensive published literature, including the experience of this group of experts, we believe that interim PET/CT assessments should be performed whenever possible to support the decision to continue or adjust treatment. 28, 29, 30, 31, 32

## Conclusion

In conclusion, we consider our analysis, conducted by a group of seven experienced hematologists employing the Delphi method, provides valuable insights into the choice between BV-AVD and ABVD as first-line therapy in advanced stage HL. Comorbid lung disease and extranodal involvement emerge as influential factors favoring BV-AVD, while considerations such as age, IPS, and mild peripheral neuropathy also impact treatment decisions.

The panel underscores that moderate-to-severe peripheral neuropathy, pregnancy, and severe drug allergies constitute absolute contraindications to BV-AVD as first-line therapy. Recognizing neuropathy as the most significant toxicity in the BV arm, the experts acknowledge its potential impact on adherence and patient management. Patients unwilling to accept the risk of neuropathy are deemed to have a relative contraindication for BV-AVD. This group suggests that BV-AVD may be a viable option for higher-risk patients and recommends considering interim PET/CT, based on results of other published clinical trials and consider a change of treatment in patients refractory to this combination. The experts highlight that under 60-year-old patients with Stage IV disease, extranodal involvement in at least two sites, and an IPS of 4–7 may derive greater benefits from BV-AVD over ABVD.

However, the introduction of BV into practice faces challenges related to healthcare system factors, including high costs, availability issues, reimbursement challenges, irregular supply, and problems with G-CSF availability.

Acknowledging the limitations, especially the absence of real-world evidence, the GATLA group proposes the creation of a prospective registry for Stage III/IV patients receiving BV-AVD. This registry aims to capture real-world data, including prognostic factors from baseline and interim PET/CT assessments.

This analysis of a randomized trial comparing BV-AVD and ABVD is composed to contribute significantly to the evolving landscape, offering practical guidance for clinicians navigating treatment options in the absence of extensive real-world evidence.

## Conflicts of interest

The author declares no conflicts of interest.
